# Locked down-locked in: experiences of families of young children with autism spectrum disorders in Delhi, India

**DOI:** 10.3389/fpubh.2024.1294538

**Published:** 2024-02-01

**Authors:** Abhipreet Kaur, Gitanjali Lall, Minal Abhilashi, Lavangi Naithani, Mamta Verma, Reetabrata Roy, Monica Juneja, Sheffali Gulati, Carol Taylor, Kathy Leadbitter, Vikram Patel, Jonathan Green, Gauri Divan

**Affiliations:** ^1^Sangath, Child Development Group, New Delhi, India; ^2^Maulana Azad Medical College Associated Lok Nayak Hospital, New Delhi, India; ^3^All India Institute of Medical Sciences, New Delhi, India; ^4^Department of Psychology and Mental Health, University of Manchester, Manchester, United Kingdom; ^5^Department of Global Health and Social Medicine, Harvard Medical School, Boston, MA, United States

**Keywords:** autism, Covid-19, family experiences, India, pandemic, qualitative

## Abstract

**Introduction:**

The onset of the COVID-19 pandemic and subsequent lockdowns in March 2020 disrupted the lives of families across India. The lockdown related restrictions brought forth a multitude of challenges including loss of employment, social isolation, school closures and financial burdens. Specifically, it also resulted in the restriction of health-care services for children with neurodevelopmental disabilities.

**Methods:**

This qualitative study was conducted as a part of a larger trial in India to understand the experiences of families of young children with autism during the pandemic. In-depth interviews were carried out with 14 caregivers residing in New Delhi, India.

**Results:**

Our findings identified pandemic and lockdown’s universal impacts on family life and financial stability stemming from job loss, business closure, and salary deductions, affecting quality of life of families. Furthermore, COVID-19 pandemic’s impact on autistic children was evident through limited access to essential services and financial challenges related service interruptions even after resumption of services. The lockdown’s novelty also affected children’s behavior, with both challenging behavioral changes and positive impacts. Primary caregivers, predominantly mothers, assumed additional responsibilities in household tasks, schooling, and therapy administration. While some these experiences were universally experienced, a few of these improved outcomes for autistic children. Despite challenges, parents expressed gratitude for their family’s safety and well-being during the difficult time.

**Discussions:**

These findings inform service provision for vulnerable families and offer implications for designing interventions such as credit schemes for families, guidance and resources for establishing and maintaining routines of children with autism, adopting flexible and adaptable approaches to service delivery, and special provisions for children with autism to be able to maintain their routines outside of home. Furthermore, the study highlights the need for comprehensive support, including educational resources and stress management counselling to empower parents in supporting essential care and routines for their children during such unprecedented times.

## 1 Introduction

In January 2020, COVID-19 emerged as a pivotal global threat and was declared a public health emergency of international concern. And the World Health Organization recommended public health measures for all nations worldwide (1). These recommendations encompassed critical measures such as social and physical distancing, meticulous hygiene practices, and the use of masks to limit the contagion of the virus and safeguard citizens (2). The Government of India documented the first case of COVID-19 on 30 January 2020 (3). As the tally of confirmed COVID-19 positive cases rose to 500, a ‘Janta Curfew’ (people’s curfew) was instituted on 22 March 2020, and on 24 March 2020, a nationwide lockdown was announced at midnight for a period of 21 days (4). This decisive measure led to comprehensive restrictions on public movement, school closures and a ban on outdoor activities. Notably, India’s lockdown has since been ranked as one of the most abrupt and stringent in the world (5).

The implementation of lockdown measures resulted in limited transportation options, constrained community interactions, and closures of both public and private sectors of employment, including education and non-emergency health care. For a substantial segment of the population, the pandemic (in particular during the devastating Delta wave in 2021) and its associated lockdowns led to severe adverse effects related to lack of accessibility to essential facilities and services, loss of family members due to the infection, job losses, mental health problems, and exacerbated financial burdens. Since educational institutions were required to shift from traditional in-person classes to online classes (6), managing household schedules became an additional challenge, especially in families where parents were working remotely while concurrently overseeing their children attending virtual classes (7).

While only a limited number of children were directly affected by the virus, they encountered several challenges due to extensive social control measures (8). The official regulations enacted as a result of lockdown measures also resulted in temporary closure of facilities offering specialized care to individuals with autism spectrum disorder, hereafter called autism. In the global context, many individuals with autism experienced a reduction in therapy hours, and in some cases, a complete absence of essential therapeutic sessions (9). It is well recognized that most children with autism need consistent routines, structured environments, and targeted therapeutic interventions to support their daily functioning. Hence, the lockdown presented several challenges including a heightened risk of worsening of behavioral symptoms (10). Empirical investigations indicated notable regression in the functioning of children with autism which was evident in domains spanning activities of daily living, language and behavioral characteristics, as well as academic and therapeutic performance (11). Additionally, a range of issues emerged after the lockdown period, including alterations in sleep patterns, attention span, concentration, limited eye contact and an increase in emotional lability, hyperactivity, and impulsivity, which were not as conspicuous prior to lockdown (11). Findings from a study in the United States of America, reported that, over half the children with autism either saw a deterioration in the symptoms associated with their pre-pandemic psychiatric diagnoses or the emergence of new psychiatric symptoms during the pandemic (12).

Even under optimal conditions, caregiving for an individual with autism can be stressful. Pre-pandemic studies indicated elevated stress levels among families with children experiencing developmental delays as compared to those with typically developing children (13). Among caregivers within this demographic, it has been seen that parents of children with autism endure heightened parenting stress (14) and psychological distress (15). The onset of COVID-19 pandemic significantly disrupted lives of families who were already grappling with parenting and psychological stress (16). These stresses not only triggered challenging behaviors, but also impacted the coping skills of both individuals and families, as well as the overall mental health and well-being of the family unit. The impact on mental health was further amplified for the caregivers of children with autism, after the onset of the lockdown (17). Notably, changes in a child’s behaviors and routines, coupled with regression in skills emerged as the significant sources of stress (18).

This study was nested in the Communication-centered Parent-mediated treatment for Autism Spectrum disorder in South Asia (COMPASS) project, launched in New Delhi on 2018 and linked to the COMPASS trial (19), which commenced just 3 months before the national lock-down in India. The aim of COMPASS trial is to evaluate the clinical-and cost-effectiveness of a parent-mediated intervention for autism designed to complement Treatment as Usual (TAU). In COMPASS, participants are randomly assigned to receive either the parent-mediated intervention in conjunction with TAU or just TAU alone. In this study, we invited primary caregivers of children with autism who had been part of the COMPASS project and had been referred from two tertiary care hospitals during the period of 2018–2021 to retrospectively share their personal experiences that transpired during the unprecedented period of COVID-19 pandemic and its associated lockdowns and explore the impact on their autistic children in particular. None of the families that are part of the study were recruited into the trial.

## 2 Materials and methods

### 2.1 Sample

The families who participated in this study were referred to the COMPASS project from specialist clinics and outpatient departments at the All India Institute of Medical Sciences (AIIMS) and Maulana Azad Medical College and associated Lok Nayak Hospital (MAMC-LNH), both tertiary care hospitals in the national capital territory of Delhi. These families were telephonically consented to participate following which, interviews were conducted telephonically over a seven-month period in 2021–2022 since face-to-face engagement with families was not feasible due to intense advocacy for social distancing and widespread fear about the virus. Data collection continued until saturation was achieved, and no novel findings or information surfaced.

### 2.2 Data collection

The data for the present study was collected through In-Depth Interviews (IDIs) which were based on a semi-structured interview guide with follow-up probes. The open ended questions in the semi-structured IDI guide were aimed at exploring the lives of families prior to the pandemic, to understand the child and family’s regular routines along with an exploration to understand the impact of the periods during the lockdowns from the first COVID-19 wave in March 2020 till the period after the second Delta wave in May 2021. The guide also covered impacts on the child’s behavior and daily routines, on caregiver’s physical and mental well-being as well as their financial security. IDIs were conducted with primary caregivers of children with autism, often over multiple contacts due to caregivers’ time constraints. These interviews were conducted by six researchers who were bilingual, fluent in both Hindi (the language of the interviews) and English (the language of the documentation), and had a minimum of a Master’s degree and experience working with families of children with autism. These researchers were associated with the COMPASS team. All researchers involved in the study were trained on qualitative analysis in general, and thematic analysis in particular. These interviews were conducted between August 2021 and February 2022.

### 2.3 Data analysis methodology

**Figure 1:**
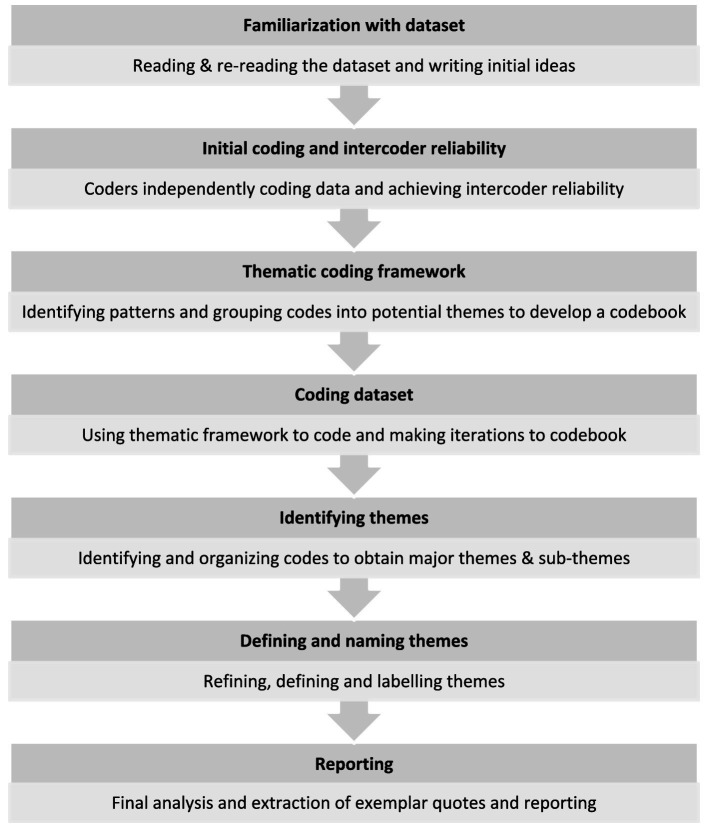
Thematic analysis process.

The IDIs were first documented using the expanded notation technique (20) in English by bilingual research team and were later analyzed using thematic analysis (21). Thematic analysis process is presented in Figure 1. AK and GD, familiarized themselves with the expanded notations of two IDIs. To enhance intercoder reliability, AK and GD then independently coded the two expanded notations line by line to capture nuances and patterns in the data. They then obtained consensus on the codes for the two interviews. This included reviewing the codes, grouping them into categories, and defining & naming them. The consensus codes were used to develop a thematic coding framework consisting of data-driven deductive codes. Using this framework, AK independently coded the expanded notations. Additionally, if any new codes emerged the codebook was revised. Upon completion of coding, the codes were reviewed to search for emerging themes. Related themes were grouped together, to obtain major themes and sub-themes. These were then reviewed, named, and defined by AK. The thematic matrix was reviewed by GD, which led to further refinement. The final stage of the process involved developing a narrative interpretation and selecting anonymized exemplar quotes which had been recorded verbatim. For reporting here, the quotes were translated from Hindi to English. The final set of themes and sub-themes obtained are described in the results section.

## 3 Results

We conducted 17 IDIs with 12 mothers and 2 fathers of children with autism (n = 14). An average of one IDI per primary caregiver was conducted, with the duration of the interview being approximately 1 h. Table 1 represents the socio-demographics characteristics of participants.

**Table 1:** Socio-demographic characteristics of the participants (*n* = 14).

Characteristics	Number (%)
Index child’s gender	
Female	11 (78.58)
Male	3 (21.42)
Primary respondent’s educational level	
Primary schooling	2 (14.28)
Secondary schooling	1 (7.14)
Higher secondary/Junior college	5 (35.71)
Graduate	4 (28.57)
Post-graduate	2 (14.28)
Primary respondent’s occupation	
Homemaker	9 (64.28)
Skilled worker	4 (28.57)
Unskilled worker	1 (7.14)
Structure of the family	
Nuclear	8 (57.14)
Multi-generational	6 (42.86)
Monthly household income (in US Dollars)	
<= 122	3 (21.43)
122–365	5 (35.71)
366–609	2 (14.29)
609–912	3 (21.43)
1219–2437	1 (7.14)
	Mean (SD)
Age of index child	8.50 (2.17)
Number of family members in house	6.21 (2.72)
Number of earning members in household	1.35 (0.5)
Number of sibling(s)	1 (0.87)

**Figure 2:**
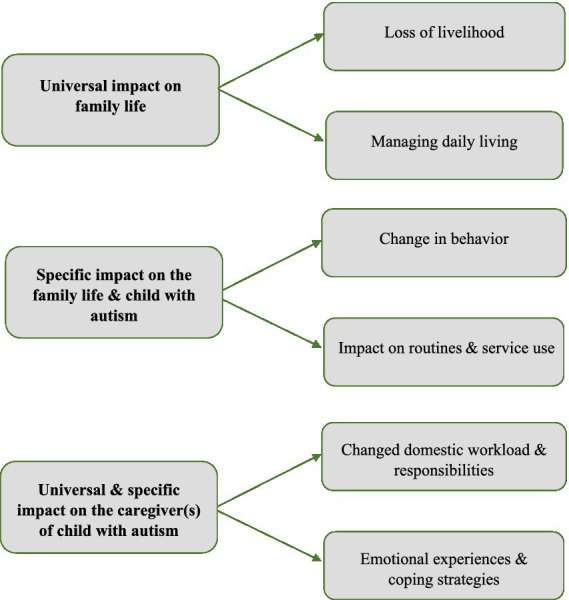
Thematic matrix developed using framework analysis.

The perspectives of the participants were categorized into three overarching themes, each comprising of two sub-themes (Refer Figure 2). The three overarching themes and sub-themes were classified into two distinct categories: Universal experiences and distinct experiences of families interviewed. While the universal experiences encapsulate themes which were common to most families during the COVID-19 pandemic, distinct experiences encompass themes highlighting experiences uniquely encountered by families of young children with autism. The caregivers interviewed often had a range of experiences both positive and negative within these themes, which are detailed below with anonymized exemplar quotes.

### 3.1 Theme 1: Universal impact on family life

This theme describes the experiences of caregivers in balancing their lives and livelihoods during the lockdowns and the intervening periods when lockdown-related restrictions were eased.

#### 3.1.1 Loss of livelihood

The overarching impact across the world and reflected in the families in this study, were the financial challenges. While three families had job security and continued to work during the lockdown, 11 participants reported financial difficulties which were attributed to inability to run their businesses (*n* = 5), salary deduction (*n* = 1) and unemployment of one or more family member(s) (*n* = 5).

*“During the second wave, we suffered major losses… these were way more than what we had faced during the first wave. Our work had completely stopped... halting our income... all the workers had gone back to their homes”* [Mother (M) 3].

*“The first phase of lockdown was manageable as we were able to save (money) but towards the end of 2020… there were some difficulties since our footwear business slowed down and savings were not enough (then) because we had to pay our staff’s salary”* (M12).

*“Her (child’s) father lost his job… her (child’s) uncle also lost his job… there were so many problems… for 8–9 months her (child’s) father remained unemployed and 8–9 months my brother-in-law… you know… because of that there were problems”* (M2).

To manage financial challenges five families restored to dipping into their savings to manage their expenses, though the long periods of lockdown meant that many families depleted these.

*“Whatever we had saved… we exhausted that during the lockdown… we had to consume it… it saddens us…”* (M2).

Additionally, there were also accounts of four of the families relying on others for financial support for their daily living expenses. This included seeking financial help from employers, friends, relatives, and Non-Governmental Organizations (NGOs).

*“We used to call the boss and say that we cannot manage our expenses, then he used to transfer money through Paytm (a digital wallet)… he helped sometimes like that…”* (M1).

*“To arrange money for rent is difficult… sometimes someone gave me some money, these were NGOs or individuals, relatives who understood our condition and wanted to help…”* [Father(F)1].

The adverse financial effects of the pandemic persisted beyond the lockdown for many families. While all the families with their own businesses were able to resume their work, they were unable to run them as effectively as they did before the pandemic due to the decreased footfall of customers in the markets and restricted physical interaction and movement due to social distancing measures. Families who relied on employers for their livelihood struggled to find new job opportunities with only three families having their earning members resume work after the lockdown, while the earning members in two families continued to face unemployment during the period of these interviews.

*“Currently, work and other activities are ongoing... The way it (business) used to be before, that’s not the case up until now, but just like everyone else’s (business) is going on, it’s going on the same way (for us) now…”* (M4).

*“There were problems getting this job as well because of my husband’s age being older than the age preferred for the job… He got his second job 8–10 months after the first lockdown ended…”* (M9).

*“…till now (since 1.5 years) I am stressed, and it’s so bad I cannot find employment yet”* (F1).

These enduring financial challenges rendered two families feeling helpless and incapacitated in fulfilling their family’s needs.

*“We try to eat only vegetables or only lentils in one meal as we cannot have both in one meal… We cannot get fruits for our children… I try to get milk for them every day at least… Earlier the child wanted a cycle but I could not get it for him… it costs Rs. 3,000 ($36.30)… he sees other children in the park and insists on getting a cycle…”* (F1).

#### 3.1.2 Managing daily living

Families described their experiences due to the financial stresses and outlined their coping strategies in managing their daily needs. A prevalent tactic (*n* = 7) was to cut down on expenses by bringing them down to a bare minimum, spending only on essentials, reducing their food intake, and either stopping or reducing the frequency of certain services like education & therapeutic services for the child.

*“We reduced our expenses like instead of half a kilogram of vegetables, we got only half of this…”* (M1).

*“Since the father did not have a job, we could only afford the therapy session once a week or once in two weeks… the therapy cost 800 rupees ($9.70) for a 45 minutes…”* (M9).

*“The household expenses that we had, we had to cut down on them… when you are working, you spend according to that… when you are not, you spend according to that… if there would not be any work then the stress would be there… everything requires money…”* (M4).

### 3.2 Theme 2: Specific impact on the family and child with autism

This theme describes the impact of the pandemic and its associated lockdowns which are specific to families who had a young child with autism. Furthermore, it also highlights the alterations in the utilization of essential services encompassing educational, therapeutic, and hospital-based services, tailored to the needs of the child with autism.

#### 3.2.1 Changes in child’s behavior

The COVID-19 pandemic and its associated lockdown had profound implications on children’s behavior. This study involved participants who reported a spectrum of experiences. Seven participants reported both positive favorable changes and increased demanding behavioral changes in their child’s behavior, while five participants noted only favorable changes during both the lockdown period and subsequent post-lockdown phase. Some positive impacts that caregivers described were observing improved communication skills, wherein children began to produce non-speech sounds, engaging in babbling, and communicating in either short or long sentences. Additionally, there were also accounts of enhanced abilities in children to express their feelings and needs to their parents.

*“…he used to speak only 2–3 words earlier like ‘Mom give water’. Staying with (other) children in the house and the neighborhood, he started speaking. The things that I could not even imagine that he would speak… now he can do all that really well…”* (M10).

*“He says that he is angry instead of hurting people… He would say ‘I want to express my anger to you because I am feeling angry…’ He is also able to give reasons as to why he is angry…”* (M9).

Caregivers suggested that being in the home environment for long periods with siblings and adult family members, resulted in an improvement in social skills like imitating others, learning through observations, and initiating social play in their children. Improved fine & gross motor skills like walking, developing a pincer grip, etc. were also observed.

*“He can now eat the food with his hand, stir with the spoon, and hold the pencils…”* (M7).

*“If there is something to eat, he would earlier eat it all… now we are seeing that he saves it for others too, as if he understands what others will have if he eats it all alone...”* (F1).

*“Now he is a little better and his vocabulary has improved…”* (M11).

*“He can now say grandpa and aunty on probing…”* (M1).

However, some participants also noted worsening of challenging behaviors, such as increased aggression & hyperactivity, increased echolalia and decreased self-expression.

*“He starts running around and gets hyperactive when he does not get what he wants… he starts shouting and whatever he finds, he puts it in his mouth… he ends up consuming random medicine if he is left unsupervised…”* (F1).

*“… after this lockdown and restrictions, the child is expressing himself less…”* (M7).

*“…there was a 70% improvement in his behavior due to his therapy, all of which became worse during the lockdown… the child’s behavior deteriorated during the COVID-19 lockdown… during the lockdown he gave me a hard time…his behavior had become somewhat different…”* (M9).

Furthermore, some participants expressed that being in the lockdown plateaued the developmental gains of their children.

*“The child’s pace of learning new skills had slowed down… Her development was delayed anyway… now it has been delayed even more… If not for COVID, she would have made more (developmental) gains…”* (F2).

#### 3.2.2 Impact on routines and service use

Another implication of the pandemic was that it led to a change in a child’s routines (*n* = 14). The sudden imposition of lockdown led to inaccessibility to various services and recreational activities like engaging in outdoor activities in the playground, playing with friends in the neighborhood, and attending schools, tuitions, or therapy centers. While talking about the leisure routines of her child, a mother shared how her child’s routines changed. She highlighted how her child’s sleeping patterns were disrupted. However, it also allowed her child to spend more time with family members, who otherwise would be at work during the day.

*“He would sleep late not before 2–3 am, and would wake up after 1–2 pm the next day… He would spend time using the phone… If he was not on the phone… he would play at home with his father or his grandfather… These would include games like ludo, playing with a ball, badminton, blocks, or even coloring... He would sit for half an hour to 45 minutes studying, sometimes even an hour…”* (M9).

As for adopting COVID-19 precautions such as wearing masks, using hand sanitizers, and social distancing, some participants reported their children found it challenging while others reported their children following these practices so religiously that when anybody in their household would step out, the child would remind them to wear mask.

*“He never forgets his mask… in fact, he reminds others to wear their mask properly...”* (M9).

Before the pandemic started, nine children were attending school or private remedial services; however, when the lockdown was imposed and in-person engagement became inaccessible, virtual schooling was accessible to just five children. Although schools tried to continue providing educational services virtually via the means of online classes and sharing classwork and homework using digital messaging apps like WhatsApp, even with these resources, the quality of education the children were receiving was severely affected.

*“In online classes children cannot understand much… for example if they are not able to understand anything in class, they can ask… and if they are still not able understand they can ask again… but they cannot do this during online classes”* (M4).

While talking about the impact of the lockdown on her child’s academics one mother shared.

*“She learnt counting from 1 to 50 (before lockdown)… during lockdown, she could not go to the private tuitions… now we have started her tuition again… she forgot what she had learned… she is being taught the same things again…”* (M2).

Nine participants reported actively needing medical consultations for comorbidities like seizures and therapeutic support like occupational therapy and speech therapy for their child prior to the pandemic. The initial cessation of services was followed by limited accessibility. While discussing about reasons for service disruptions during the lockdown, families attributed inaccessibility to hospital and therapy centers, absence of online alternatives and heighted concerns related to COVID-19 safety measures as some of the reasons for it.

*“…speech therapy had to be stopped since no therapist was available at the center…”* (M9).

*“My child’s therapy also had to be paused. We managed getting sessions (speech and physiotherapy) at home but considering safety, (we) again stopped the* sessions…” (M12).

While talking about hospital-based services, the participants also highlighted that all the special pediatric wards within government hospitals were repurposed into COVID-19 treatment facilities thus rendering hospital-based services completely inaccessible with a resultant compromise in the care of their child.

*“My boy… he was experiencing seizures… then we took him to the hospital… the whole hospital was turned into COVID hospital… The entire hospital was seeing Corona patients only... His medicines had to be changed, because of seizures he was shaking and he passed stool and urine too... Recently, when we went to the doctor, they asked us why did you come so late…”* (M1).

*“Then lockdown happened and they (the tertiary care center) refused to conduct check-ups… they told us to keep the child at home…”* (F1).

*“We were forced to drop out of treatment due to the pandemic-related containment and social distancing measures…”* (F2).

Three families managed to receive medical consultations from local clinics, while five families were able to establish at least partial therapeutic support for their child during the initial months of the lockdown.

*“There are small clinics... we used to get medicines from there.”* (M4).

*“The child has started visiting the therapy center every Friday since the end of last year* (i.e.*, December 2020*)*…”* (M6).

Furthermore, following the relaxations in COVID-19 related restrictions four families were able to reinstate therapeutic and medical support for their children.

*“Also R’s (child’s) sleep had reduced so we visited the hospital on the 5th November 2021 where they prescribed one medicine… In one week’s time R was calmer…”* (M12).

*“Speech therapy was resumed in August 2021 with a new therapist at the same center. This therapist has been focusing more on comprehension skills and it has been very useful…”* (M9).

As the impact of service cessation unfolded for caregivers one participant realized the importance of therapies and it encouraged them to seek therapeutic support consistently for their child after the COVID-19-related restrictions were eased.

### 3.3 Theme 3: Universal and specific impact on the caregiver(s) of child with autism

This theme describes the practical and emotional impacts of the pandemic and its associated lockdown on the primary caregivers as well as the coping strategies used by them during that period.

#### 3.3.1 Changed domestic workload and responsibilities

The lockdown resulted in all members of a family being confined to their homes, while children were meant to be supported with virtual services and school lessons. This resulted in an increase in the workload for the majority of mothers which was universal across households. However, the lockdown led to new caregiver responsibilities such as teaching their child and practicing therapeutic skills at home which was specific to families of children with autism. Half the mothers reported an increased workload at home, while two reported decreased workload and one reported no change.

*“There was an increase in workload as my husband was at home… He would demand for different dishes, so I would keep on making something or the other for him…”* (M2).

*“…my responsibilities decreased during COVID… I did not have to take my child for any services. I was also able to interact with relatives more…”* (M10).

*“We are just 4 members… only his father used to go (out to work) that is why there was no change for us… neither there was an increase… nor was there a decrease…”* (M4).

Increased child focused responsibilities during the lockdown included practicing therapeutic skills (*n* = 7), assisting the child with classwork and homework (*n* = 4), and playing and interacting with their child for longer periods (*n* = 3). These experiences were particularly relevant to families of children with autism.

*“We would make him sit in front of the mirror and then we would make sounds while also exaggerating our lip movements… also do actions so that he can try and copy…”* (M3).

*“The child needed support with his studies, for example, copying the homework sent by WhatsApp …”* (M8).

Additionally, a subset of participants reported that the restrictions imposed during the pandemic facilitated enhanced familial support for mothers across several domestic responsibilities including helping the child with academics, practicing home program skills, cooking, and cleaning. Concurrently, respondent mothers also reported that the lockdown allowed fathers with increased opportunities to engage with their children. Although, these experiences were universal to all families, they were significantly associated with improved outcomes among children with autism. These improvements were attributed to the increased and sustained social engagement children experienced with their family members.

*“Everybody at home is very nice… my brother-in-law and husband used to cook… they used to cook something or the other…”* (M2).

*“Whatever was done online (for school)… my child would do it online and since his father used to be there… whenever he had time, he also used to help with that (academics)… It happened only in the lockdown… earlier his father used to go to work… so, it could not be done… I stay at home, so I used to take care of it…”* (M4).

*“My younger son is dusting… After observing him, my elder son has started asking ‘mom can I help you with the dishes?’ While I am cooking, they would come to me and say ‘I will also do it… learn it’…”* (M7).

Although majority of the family members made efforts to assist mothers, there were also instances particularly around home programs where mothers did not receive any support. This observation was particularly relevant to families of children with autism.

*“…however, nobody knew how to practice therapeutic skills with the child… the mother knew it… other family members could not help with it…”* (F2).

#### 3.3.2 Emotional experiences and coping strategies

Uncertainty, fear, and emotional distress were the common emotions described by participants. These distressing emotional experiences were triggered by financial problems, family dynamics, inability to provide essential services to their child, fear of contracting the virus, and fear of job loss among the breadwinners in the family.

*“We dwell within ourselves, lost in our own thoughts and worries. Now, what should we do?! The body has also become weak...”* (F1).

*“…that time was very stressful… we had just got our child admitted to a school… we were hopeful that if he would go to this school, we would see changes in him… he’ll improve… we’ll see it…”* (M4).

*“Nobody had tested positive (with COVID)… there were so many kids who lost their parents… there were so many things… we never even imagined something like this… there was no value for a person’s life… people were busy making black money (undisclosed income)… were selling oxygen cylinders in black (illegal sales)…”* (M3).

*“There was, however, a perpetual fear of job loss that served as a great source of stress during the pandemic… Every morning after waking up… we would pray to God to protect (my husband’s) job…* (M6).

All families reported emotional distress due to inability to seek essential services like schooling, therapies, etc. for the child.

*“The child used to ask, do we again have to remain inside our house? Do we need to stop going into the playground again? If Corona comes back then what will happen?”* (M7).

*“I was worried about my child… neither could I take her anywhere (for therapy) nor I was able to consult… I could not talk to anybody (the therapist)…* (M2).

*“We will not be able to make up for school… the things children could do in school, they are not able to do them at home… and what the tuition teacher teaches… whatever is taught online… both of them are different… so, they were not able to comprehend that much…”* (M4).

*“I am still very upset that he could not start his schooling… he needs it very much… It is his critical time to learn things… we could not do anything… this time has been wasted…”* (M3).

On the other hand, five participants also described experiencing positive emotions, especially gratitude. They felt thankful for being safe during the pandemic with their family members at their homes, and also acknowledged their privileges.

*“The most important thing was that we had a house of our own… it was not rented… if it would have been rented then it would have been problematic for us… whether you eat or not… but you have to pay the rent…”* (M2).

*“All that mattered to us was that all our family members were safely at home… we could eat the way we wanted to at home… but there were so many people who did not have a home here (in Delhi)… who were worried about food and were uncertain whether they’ll get something to eat or not… we used to get stressed because of that not because of the financial loss we had…”* (M3).

Seeking support from family, social networks, and religious practices emerged as the key coping strategies (*n* = 9). Along with these, some used strategies such as avoidance (*n* = 2), distraction (*n* = 2), and positive thinking (*n* = 1).

*“It was frustrating at times but because of each other, we could manage ourselves…”* (M7).

*“Since it was a time where nobody could step outside and to ensure family’s safety, we would take both the child and his cousin out for car rides so that they can be outside for a while…* (M3).

*“We used to pray to god that everything gets fine… it was a bad time which has passed… we just hope that it does not come back…”* (M2).

*“I find my mother and husband as my two pillars of strength. Whenever there are thoughts of losing patience, my husband and my mother are there to lend ears. I feel good after talking to them”* (M6).

## 4 Discussion

We describe the findings of a qualitative study which aimed to explore the experiences of a group of families of autistic children during the COVID-19 pandemic and the associated lockdowns in New Delhi, India, a Low-and Middle-Income Country (LMIC). The findings from our study, derived from 17 IDIs, highlight a significant impact of COVID-19 and the associated lockdowns on families. The difficulties faced by families as they grappled with financial challenges and encountered hurdles in maintaining their daily lives, are evident in the data from our sample. It is important to note that these experiences based on the interviews conducted in this study resonate with similar situations reported across the world, including studies conducted in various nations including several High Income Countries (HIC), like United States of America (22–24). The effects on families with children with autism were multifaceted, with primary caregivers reporting both positive and adverse alterations in their child’s behavior as well as autism related symptoms. Furthermore, there were specific repercussions on children’s daily routines and access to services. Primary caregivers, in the majority of instances, mothers, reported a significant shift in their roles and responsibilities within the household during the pandemic. Additionally, these primary caregivers shared their emotional experiences and the coping strategies that they employed. These experiences were both universal (25) and specific to families of children with autism within the wider parenting experience of caregivers of children with disabilities during the pandemic (26). Importantly, these shared and unique challenges transcended geographical boundaries and were experienced by several families across low-, middle-and high-income country settings (25, 27). We found that the majority of families within our sample received their main financial earnings from private employment or their own businesses both of which were impacted by the lockdown. These financial impacts on the breadwinner had implications on the quality of lives of all family members in managing their daily requirements, aligning with the situation across the country in general, which showed an increase in the unemployment rate in both urban and rural settings (28–30).

The pandemic and associated lockdown came with school closures and a switch to online modes of learning (31–34). Our findings showed that the learning loss which children experienced due to school closure was acutely felt in children with autism. This impact on learning due to school closures was compounded by interruption to regular therapy services, which is often directed at supporting engagement in the classroom setting and remediation, making the autistic children vulnerable to learning loss (9, 35, 36).

The lockdown presented a relatively new situation for families, which impacted children’s behavior (37). While these findings of an increase in challenging behavior, including aggression and hyperactivity align with the previous research conducted in both LMICs and HICs, generalization of these trends may be constrained given the small sample size (11, 38–43). A preference for circumscribed and regular routines in autistic individuals is a universal characteristic (44), its disruption during the early days of the lockdown with caregivers reporting sleep disturbances, hyperactivity and aggression, has been a noted in other research (39, 41, 42, 45). Caregivers across different socio-economic contexts reported similar challenges, thus indicating a shared experience for autistic children, regardless of the income levels of their respective countries. An unexpected outcome was a subset of caregivers who reported positive impacts on their children’s behavior and social communication skills. These changes which have been postulated to be due to ‘reduced travelling, exposure to unfamiliar environments and more time spent socializing with family members, at home, at a relaxed self-paced schedule’ (42) were also described by some of the caregivers in this study. Mothers in our study felt that the presence of fathers at home, often missing in the context of routine of urban working family life, may have helped their child, especially boys, by being role models. The presence of siblings due to school closure may have also added to the opportunities of social interactions in a safe setting. Mothers reported spending increased time with their child, playing with them and practicing home program skills when these were available.

Studies globally have found both improvement (17) and deterioration in behavior (38, 41) of young children with autism as a result of the pandemic. In terms of COVID-specific behavior such as wearing masks, regular hand washing etc., our study yielded mixed findings. While some children embraced the precautions, others found it difficult to adhere to them, and this may be due to underlying sensory sensitivities or a difficulty in adapting to novel routines (46, 47).

Mothers, as the primary caregivers of their children, were burdened with the additional responsibilities of household chores, schooling and therapy administration. Our findings are in line with extant research done in HICs which has indicated that during the pandemic, the additional responsibilities of work within homes fell on women (48). Significantly, the majority of caregivers, encompassing various economic context, reported increased levels of stress during this time, due to financial distress, increased workload, fear of possible infection and most importantly, the inability to access academic, medical and therapeutic support for their child (43, 49). Despite challenges most caregivers in retrospect were able to express gratitude for having survived the pandemic without any major losses. A key strength of our study was the use of IDI which allowed the collection of rich data, describing the experiences of the caregivers. Participants selected were already engaged with the COMPASS project thus increasing the possibility of participation given the already established levels of trust. Conducting interviews over the telephone increased flexibility of participation, allowing the participants to complete the interview in more than one session. This also is one of the study limitations, since it did not allow for picking up the nuances of body language which may have been possible within an in-person setting. Furthermore, considering the interval of over a year during the initial COVID-19 lockdown in conducting these interviews, potential recall bias is a concern. Additionally, conducting IDIs over multiple telephone calls led to interruptions in the flow of conversation. Finally, given the limited sample size and restricted geography of the study, we cannot generalize these findings to other contexts in India.

This qualitative study contributes to the limited but steadily growing literature on the experiences of families with young children with autism during the COVID-19 pandemic, especially within the context of countries like India. These findings pose several implications for service provision and design of interventions tailored to cater the unique challenges faced by families of children with autism during such unprecedented times. Firstly, though the Government of India, had instituted in-kind or cash transfers for low income families, none of the respondents received this, implying that approaches like the United Kingdom’s Universal Credit Scheme may be a better method to support the universal distress of a pandemic shock (50). Secondly, restricted access to health services has been described across contexts (51), and though many clinical centers in New Delhi, reached out to families, none of the families were provided with guidance and resources for establishing and maintaining structured routines for their children which we know could have mitigated a negative impact of behavioral challenges. Furthermore, disruptions due to the pandemic highlighted a critical need for service providers to adopt a flexible and adaptable approach to service delivery to ensure uninterrupted delivery of medicines, as well as therapeutic services, thereby allowing uninterrupted access to these essential services. Incorporating remote and virtual intervention modalities like tele-therapy, can be a vital strategy to support families. Additionally, adequate support is required to equip parents with tools essential for effectively supporting educational needs of their child, which requires a collaboration between educational institutions, special education services and parents so as to develop effective remote learning plans. An important constraint many of the families experienced was the restriction of movements and being ‘locked-in’ which exacerbated the behavioral problems in their child. A legal case in the United Kingdom, resulted in a change in the emergency pandemic restrictions and supported individuals with intellectual disabilities and autism to leave their homes for exercise more than once a day (52). This nuanced understanding of the needs of specific communities should be part of guidelines that should be developed to support disaster preparedness in the future. Moreover, findings from this study highlighted a compelling need to focus on caregiver well-being thus necessitating the implementation of stress management counselling services and resources. These findings, when integrated into service provisions and interventions, offer a comprehensive approach to addressing the unique challenges faced by children with autism and their families during periods of crisis, thereby promoting their holistic well-being.

## Data availability statement

The raw data supporting the conclusions of this article will be made available by the authors, without undue reservation.

## Ethics statement

The studies involving humans were approved by Sangath Institutional Review Board. This study was conducted in accordance with the local legislation and institutional requirements. The participants provided their verbal informed consent to participate in this study.

## Author contributions

AK: Data curation, Investigation, Formal analysis, Writing – original draft, Writing – review & editing. GL: Data curation, Investigation, Writing – original draft. MA: Data curation, Investigation, Writing – original draft, Writing – review & editing. LN: Data curation, Investigation, Writing – original draft. MV: Data curation, Investigation, Writing – original draft. RR: Writing – review & editing. MJ: Resources, Writing – review & editing. SG: Resources, Writing – review & editing. CT: Writing – review & editing. KL: Conceptualization, Writing – review & editing. VP: Conceptualization, Funding acquisition, Writing – review & editing. JG: Funding acquisition, Writing – review & editing. GD: Conceptualization, Formal analysis, Funding acquisition, Supervision, Writing – review & editing.

## References

[1] WHO. (2020). COVID-19 public health emergency of international concern (PHEIC) Global research and innovation forum. Available at: https://www.who.int/publications/m/item/covid-19-public-health-emergency-of-international-concern-(pheic)-global-research-and-innovation-forum

[2] Levante A Petrocchi S Bianco F Castelli I Colombi C Keller R. Psychological impact of COVID-19 outbreak on families of children with autism Spectrum disorder and typically developing peers: an online survey. Brain Sci. (2021) 11:808. doi: 10.3390/brainsci11060808, PMID: 34207173 PMC8235600

[3] Andrews MA Areekal B Rajesh KR Krishnan J Suryakala R Krishnan B. First confirmed case of COVID-19 infection in India: a case report. Indian J Med Res. (2020) 151:490–2. doi: 10.4103/ijmr.IJMR_2131_20, PMID: 32611918 PMC7530459

[4] Gettleman J. Schultz K. (2020). Modi orders 3-week Total lockdown for all 1.3 billion Indians. The New York Times. Available at: https://www.nytimes.com/2020/03/24/world/asia/india-coronavirus-lockdown.html

[5] Choudhury C. (2020). India’s COVID-19 lockdown is among the strictest in the world but has yet to slow the spread. CBC News. Available at: https://www.cbc.ca/news/world/india-lockdown-covid-1.5534551

[6] Macwan G. (2020). Delhi government schools to hold interactive online classes in April 2020 Amid COVID-19 lockdown. Jagranjosh.Com. Available at: https://www.jagranjosh.com/news/delhi-government-schools-to-hold-interactive-online-classes-from-1st-april-2020-amid-covid-19-lockdown-153428

[7] Evans S Mikocka-Walus A Klas A Olive L Sciberras E Karantzas G. From “it has stopped our lives” to “spending more time together has strengthened bonds”: the varied experiences of Australian families during COVID-19. Front Psychol. (2020) 11:588667. doi: 10.3389/fpsyg.2020.588667, PMID: 33192922 PMC7606874

[8] Raman S Harries M Nathawad R Kyeremateng R Seth R Lonne B. Where do we go from here? A child rights-based response to COVID-19. BMJ Paediatrics Open. (2020) 4:e000714. doi: 10.1136/bmjpo-2020-000714, PMID: 32577537 PMC7299026

[9] Eshraghi AA Li C Alessandri M Messinger DS Eshraghi RS Mittal R. COVID-19: overcoming the challenges faced by individuals with autism and their families. Lancet Psychiatry. (2020) 7:481–3. doi: 10.1016/S2215-0366(20)30197-8, PMID: 32445682 PMC7239613

[10] Panda PK Gupta J Chowdhury SR Kumar R Meena AK Madaan P. Psychological and behavioral impact of lockdown and quarantine measures for COVID-19 pandemic on children, adolescents and caregivers: a systematic review and Meta-analysis. J Trop Pediatr. (2020) 67:fmaa122. doi: 10.1093/tropej/fmaa122, PMID: 33367907 PMC7798512

[11] Kaur R Boobna T Kallingal P. Effect of Covid-19 lockdown on indian children with autism. Res Dev Disabil. (2022) 125:104230. doi: 10.1016/j.ridd.2022.104230, PMID: 35367807 PMC8964314

[12] Vasa RA Singh V Holingue C Kalb LG Jang Y Keefer A. Psychiatric problems during the COVID-19 pandemic in children with autism spectrum disorder. Autism Res: Official J Int Society for Autism Res. (2021) 14:2113–9. doi: 10.1002/aur.2574, PMID: 34231323 PMC8420610

[13] Baker BL Blacher J Crnic KA Edelbrock C. Behavior problems and parenting stress in families of three-year-old children with and without developmental delays. American J Mental Retardation: AJMR. (2002) 107:433–44. doi: 10.1352/0895-8017(2002)107<0433:BPAPSI>2.0.CO;2, PMID: 12323068

[14] Hayes SA Watson SL. The impact of parenting stress: a meta-analysis of studies comparing the experience of parenting stress in parents of children with and without autism spectrum disorder. J Autism Dev Disord. (2013) 43:629–42. doi: 10.1007/s10803-012-1604-y, PMID: 22790429

[15] Estes A Munson J Dawson G Koehler E Zhou X-H Abbott R. Parenting stress and psychological functioning among mothers of preschool children with autism and developmental delay. Autism: Int J Res Prac. (2009) 13:375–87. doi: 10.1177/1362361309105658, PMID: 19535467 PMC2965631

[16] Manning J Billian J Matson J Allen C Soares N. Perceptions of families of individuals with autism Spectrum disorder during the COVID-19 crisis. J Autism Dev Disord. (2021) 51:2920–8. doi: 10.1007/s10803-020-04760-5, PMID: 33090358 PMC7578441

[17] Lugo-Marín J Gisbert-Gustemps L Setien-Ramos I Español-Martín G Ibañez-Jimenez P Forner-Puntonet M. COVID-19 pandemic effects in people with autism Spectrum disorder and their caregivers: evaluation of social distancing and lockdown impact on mental health and general status. Res Autism Spectr Disord. (2021) 83:101757. doi: 10.1016/j.rasd.2021.101757, PMID: 33649707 PMC7904459

[18] BharatRUzainaNiranjanSYadavTNewmanSMarriottJ. Autism Spectrum disorder in the COVID 19 era: new challenges - new solutions. Indian Pediatr. (2021) 58:890–1. doi: 10.1007/s13312-021-2314-3, PMID: 34183470

[19] Roy R Leadbitter K Shields G Taylor C Aldred C Juneja M. A randomised controlled trial of clinical and cost-effectiveness of the PASS plus intervention for young children with autism spectrum disorder in New Delhi, India: study protocol for the COMPASS trial. Trials. (2023) 24:667. doi: 10.21203/rs.3.rs-2353521/v1 37828540 PMC10571330

[20] Halcomb EJ Davidson PM. Is verbatim transcription of interview data always necessary? Appl Nurs Res. (2006) 19:38–42. doi: 10.1016/j.apnr.2005.06.001 16455440

[21] Braun V Clarke V. Using thematic analysis in psychology Qual Res Psychol. (2006) 3:77–101. doi: 10.1191/1478088706qp063oa

[22] Andrade C Gillen M Molina JA Wilmarth MJ. The social and economic impact of Covid-19 on family functioning and well-being: where do we go from here? J Fam Econ Iss. (2022) 43:205–12. doi: 10.1007/s10834-022-09848-x, PMID: 35669394 PMC9136200

[23] NEFE. (2020). Nearly 9 in 10 say COVID-19 crisis is causing financial stress. Available at: https://www.nefe.org/news/2020/04/survey-covid-19-crisisi-causing-financial-stress.aspx

[24] Rodrigues M Silva R Franco M. COVID-19: financial stress and well-being in families. J Fam Issues. (2023) 44:1254–75. doi: 10.1177/0192513X211057009, PMID: 37064997 PMC10090962

[25] Connor J Madhavan S Mokashi M Amanuel H Johnson NR Pace LE. Health risks and outcomes that disproportionately affect women during the Covid-19 pandemic: a review. Soc Sci Med. (2020) 266:113364. doi: 10.1016/j.socscimed.2020.113364, PMID: 32950924 PMC7487147

[26] Aishworiya R Kang YQ. Including children with developmental disabilities in the equation during this COVID-19 pandemic. J Autism Dev Disord. (2021) 51:2155–8. doi: 10.1007/s10803-020-04670-6, PMID: 32816170 PMC7438977

[27] Penna AL de Aquino CM Pinheiro MSN Nascimento RLF Farias-Antúnez S Araújo DABS. Impact of the COVID-19 pandemic on maternal mental health, early childhood development, and parental practices: a global scoping review. BMC Public Health. (2023) 23:1–26. doi: 10.1186/s12889-023-15003-4, PMID: 36823592 PMC9950022

[28] Akhter Ali M Kamraju M. A study on impact of COVID-19 pandemic on unemployment in India. Re-Imagining the New Normal-The Transformational Lens of COVID. (2020) 19:50–61.

[29] Ghosh A Nundy S Mallick TK. How India is dealing with COVID-19 pandemic. Sensors Int. (2020) 1:100021. doi: 10.1016/j.sintl.2020.100021, PMID: 34766039 PMC7376361

[30] Paul B Patnaik U Murari KK Sahu SK Muralidharan T. The impact of COVID-19 on the household economy of India. Indian J Labour Econ: Q J Indian Society of Labour Econ. (2021) 64:867–82. doi: 10.1007/s41027-021-00352-8, PMID: 34803248 PMC8596369

[31] Jain A Ahmed N Mahour P Agarwal V Chandra K Shrivatav NK. Burden of care perceived by the principal caregivers of autistic children and adolescents visiting health facilities in Lucknow City. Indian J Public Health. (2019) 63:282–7. doi: 10.4103/ijph.IJPH_366_18, PMID: 32189645

[32] Jena PK. Impact of pandemic COVID-19 on education in India. Int J Current Res. (2020) 12:12582–6. doi: 10.31235/osf.io/2kasu

[33] Mahapatra A Sharma P. Education in times of COVID-19 pandemic: academic stress and its psychosocial impact on children and adolescents in India. Int J Soc Psychiatry. (2021) 67:397–9. doi: 10.1177/0020764020961801, PMID: 32972291

[34] Tarkar P. Impact of Covid-19 pandemic on education system. Int J Advanced Sci Technol. (2020) 29:3812–4.

[35] Baweja R Brown SL Edwards EM Murray MJ. COVID-19 pandemic and impact on patients with autism Spectrum disorder. J Autism Dev Disord. (2022) 52:473–82. doi: 10.1007/s10803-021-04950-9, PMID: 33689088 PMC7943706

[36] Musa S Dergaa I Mansy O. The puzzle of autism in the time of COVID-19 pandemic: ‘light it up blue’. Psychology (Savannah, Ga). (2021) 58:1861–73.

[37] Tokatly Latzer I Leitner Y Karnieli-Miller O. Core experiences of parents of children with autism during the COVID-19 pandemic lockdown. Autism: Int J Res Prac. (2021) 25:1047–59. doi: 10.1177/1362361320984317, PMID: 33435701

[38] Colizzi M Sironi E Antonini F Ciceri ML Bovo C Zoccante L. Psychosocial and behavioral impact of COVID-19 in autism Spectrum disorder: an online parent survey. Brain Sci. (2020) 10:341. doi: 10.3390/brainsci10060341, PMID: 32503172 PMC7349059

[39] Di Renzo M Bianchi di Castelbianco F Vanadia E Petrillo M D’Errico S Racinaro L. Parent-reported Behavioural changes in children with autism Spectrum disorder during the COVID-19 lockdown in Italy. Contin Educ. (2020) 1:117–25. doi: 10.5334/cie.20 PMC11104382 38774533

[40] Franz K Kelly ME. The Behavioural outcomes of children with autism Spectrum disorder and other developmental disabilities as perceived by parents during the COVID-19 lockdown. Disabilities. (2021) 1:347–60. doi: 10.3390/disabilities1040024

[41] Huang S Sun T Zhu Y Song S Zhang J Huang L. Impact of the COVID-19 pandemic on children with ASD and their families: an online survey in China. Psychol Res Behav Manag. (2021) 14:289–97. doi: 10.2147/PRBM.S293426, PMID: 33692639 PMC7939504

[42] Kaku SM Chandran S Roopa N Choudhary A Ramesh J Somashekariah S. Coping with autism during lockdown period of the COVID-19 pandemic: a cross-sectional survey. Indian J Psychiatry. (2021) 63:568–74. doi: 10.4103/indianjpsychiatry.indianjpsychiatry_344_21, PMID: 35136254 PMC8793703

[43] Mutluer T Doenyas C Aslan Genc H. Behavioral implications of the Covid-19 process for autism Spectrum disorder, and individuals’ comprehension of and reactions to the pandemic conditions. Front Psych. (2020) 11:561882. doi: 10.3389/fpsyt.2020.561882, PMID: 33304279 PMC7701051

[44] Hyman SL Levy SE Myers SM Council on Children With Disabilities, Section on Developmental and Behavioral Pediatrics. Identification, evaluation, and Management of Children with Autism Spectrum Disorder. Pediatrics. (2020) 145:e20193447. doi: 10.1542/peds.2019-3447 31843864

[45] Eshraghi AA Cavalcante L Furar E Alessandri M Eshraghi RS Armstrong FD. Implications of parental stress on worsening of behavioral problems in children with autism during COVID-19 pandemic: ‘the spillover hypothesis’. Mol Psychiatry. (2022) 27:1869–70. doi: 10.1038/s41380-021-01433-2, PMID: 35064235 PMC8780050

[46] Parenteau CI Bent S Hossain RL Hossain B Chen Y Widjaja F. The experience of parents of children with autism spectrum disorder during the COVID-19 pandemic: A qualitative analysis. J Am Acad Child Adolesc Psychiatry. (2020) 59:S251. doi: 10.1016/j.jaac.2020.08.411

[47] Tamon H Itahashi T Yamaguchi S Tachibana Y Fujino J Igarashi M. Autistic children and adolescents with frequent restricted interest and repetitive behavior showed more difficulty in social cognition during mask-wearing during the COVID-19 pandemic: a multisite survey. BMC Psychiatry. (2022) 22:608. doi: 10.1186/s12888-022-04249-8, PMID: 36104779 PMC9471034

[48] Del Boca D Oggero N Profeta P Rossi M. Women’s and men’s work, housework and childcare, before and during COVID-19. Rev Econ Househ. (2020) 18:1001–17. doi: 10.1007/s11150-020-09502-1, PMID: 32922242 PMC7474798

[49] Yılmaz B Azak M Şahin N. Mental health of parents of children with autism spectrum disorder during COVID-19 pandemic: a systematic review. World journal of. Psychiatry. (2021) 11:388–402. doi: 10.5498/wjp.v11.i7.388, PMID: 34327131 PMC8311509

[50] Policy Responses to COVID19. (2021). IMF. Available at: https://www.imf.org/en/Topics/imf-and-covid19/Policy-Responses-to-COVID-19

[51] Pujolar G Oliver-Anglès A Vargas I Vázquez M-L. Changes in access to health services during the COVID-19 pandemic: a scoping review. Int J Environ Res Public Health. (2022) 19:1749. doi: 10.3390/ijerph19031749, PMID: 35162772 PMC8834942

[52] Walker A. (2020). UK coronavirus rules relaxed for people with autism and learning disabilities. The Guardian. Available at: https://www.theguardian.com/world/2020/apr/14/uk-coronavirus-rules-autism-learning-disabilities-lockdown

